# The Effects of Backward Adjustable Thoracic Support in Wheelchair on Spinal Curvature and Back Muscle Activation for Elderly People

**DOI:** 10.1371/journal.pone.0113644

**Published:** 2014-11-26

**Authors:** Chun-Ting Li, Yen-Nien Chen, Chih-Han Chang, Kuen-Horng Tsai

**Affiliations:** 1 Institute of Biomedical Engineering, National Cheng Kung University, Tainan, Taiwan; 2 Metal Industries Research & Development Centre, Kaohsiung, Taiwan; 3 Graduate Institute of Mechatronic System Engineering, National University of Tainan, Tainan, Taiwan; University of Rome Foro Italico, Italy

## Abstract

**Objectives:**

To investigate the effects of backward adjustable thoracic support on spinal curvature and back muscle activation during wheelchair sitting.

**Methods:**

Twenty elderly people were recruited for this study. The backward adjustable thoracic support sitting posture was compared with the slumped, normal, and lumbar support sitting postures. Spinal curvatures (pelvic, lumbar, and thoracic angles) and muscle activations of 4 back muscles on both sides (maximal voluntary isometric contraction of the lumbar multifidus, lumbar erector spinae, iliocostalis lumborum pars thoracis, and thoracic erector spinae at T9) were measured and compared between the different sitting postures using one-way analysis of variance with repeated measures.

**Results:**

The backward adjustable thoracic support sitting posture showed a relatively neutral pelvic tilt (−0.32±4.80°) when compared with the slumped (22.84±5.27°) and lumbar support (−8.97±3.31°) sitting postures (*P*<0.001), and showed relatively higher lumbar lordosis (−23.38±6.50°) when compared with the slumped (14.77±7.83°), normal (0.44±7.47°), and lumbar support (−16.76±4.77°) sitting postures (*P*<0.05). It also showed relatively lower back muscle activity when compared with the normal and lumbar support sitting postures (*P*<0.05).

**Conclusions:**

The backward adjustable thoracic support sitting concept was suggested because it maintains a more neutral pelvic tilt, higher lumbar lordosis, and lower back muscle activation, which may help maintain a better sitting posture and reduce the risk of back pain.

## Introduction

Elderly adults with lower limb disorders must use wheelchairs to improve their mobility. Back pain is a common complication among wheelchair users [1], [2]. Clinical observation shows that wheelchair-bound elderly adults often exhibit slumped sitting, which increases the risk of back pain [2]. Choosing a suitable seating system is crucial for wheelchair-bound elderly adults because the seating system may influence spinal curvature and back muscle activation, which are critical mechanisms of back pain [3]–[8].

When sitting, maintaining lumbar lordosis can reduce the stress on the intervertebral discs and the load on back muscles [9]. Because of the lumbar-pelvic rhythm, lumbar lordosis occurs with anterior pelvic tilt, whereas lumbar kyphosis occurs with posterior pelvic tilt [10]. In addition, pelvic tilt is correlated with hamstring tightness. Anterior pelvic tilt increases hamstring tightness, whereas posterior pelvic tilt reduces hamstring tightness [11], [12]. Therefore, lumbar curvature, pelvic tilt, back muscle activation, and hamstring tightness are correlated and all influence the maintenance of sitting posture.

Regarding seating systems, the most frequently used system in the clinical care is the standard sling seat and back upholstery, which pose limitations on the maintenance of optimal posture [2]. Previous studies have indicated that reclined backrest, forward tilted seat, and lumbar support can help reduce lumbar load [13], [14]. However, a reclined backrest may disrupt the functional sitting posture and may cause the user to slide forward, thus generating an undesirable sitting posture [13], [14]. Using a forward tilted seat risks causing the user to slide forward when moving the wheelchair backward, thereby increasing the danger of using the wheelchair at slopes [17]. Lumbar support can maintain lumbar lordosis. However, to enable sufficient lumbar lordosis, anterior pelvic tilt and hamstring tightness are inevitable, which subsequently influence the comfort of posture maintenance [2], [10]–[12]. Clinical observations have shown that numerous wheelchair users exhibit undesirable sitting posture despite using lumbar support. Although a number of seating systems have been promoted [18], [19], few studies have focused comprehensively on the aforementioned problems.

**Figure 1:**
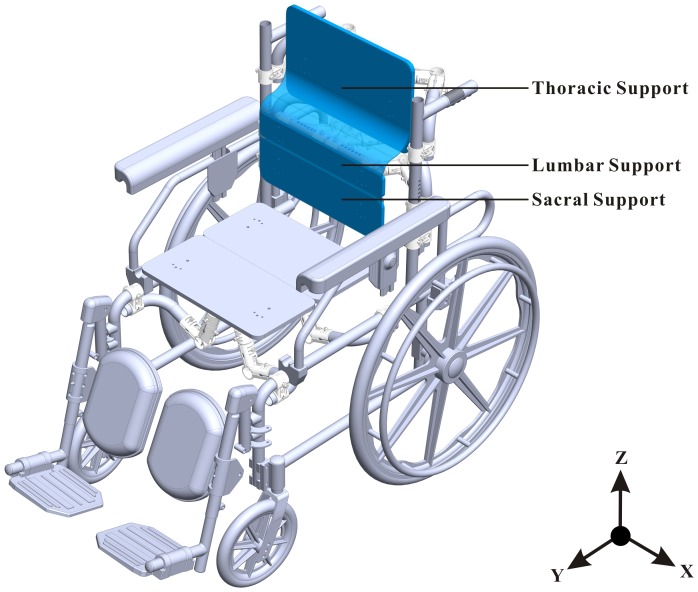
The experimental wheelchair. The back support components included the adjustable sacral, lumbar, and thoracic supports. Each component can be adjusted in the y-axis translation, the z-axis translation, and the x-axis rotation. The seat height, the seat depth, and the pedal length can also be adjusted. The current setting is backward adjustable thoracic support.

To solve the problems, we proposed a new sitting posture concept, the backward adjustable thoracic support (BTS) posture, as shown in Figure 1. We assume that the BTS posture can maintain neutral pelvic tilt and lumbar lordosis through lower back muscle activation at upright sitting posture and indirectly alleviate hamstring tightness and stress on the intervertebral disc. The purpose of the present study was to evaluate the spinal curvature and back muscle activation when using the BTS in the elderly population. The hypothesis was as follows: When the thoracic support is backward adjusted, it results in a rather neutral pelvic tilt, increased lumbar lordosis, and low back muscle activity. In particular, the BTS was compared with the slumped sitting (SS) posture, normal sitting (NS) posture, and lumbar support sitting (LSS) posture.

## Methods

### Participants

Twenty elderly men (age, 70.20±3.71 years old; weight, 69.32±10.49 kg; height, 165.48±5.96 cm; body mass index, 25.26±3.28 kg/m^2^) voluntarily participated in the present study. During the recruitment period, we screened out those with identifiable musculoskeletal disorders, movement disorders, and spinal pathology requiring treatment during the preceding 6 months. All the participants read and signed an informed consent form that explained the objectives and experimental protocol. This study was approved by the Institutional Review Board of National Cheng Kung University Hospital.

### Experimental Wheelchair

An experimental wheelchair was designed for the study (Figure 1). The back support components were designed for supporting the sacral, lumbar, and thoracic regions. Each component can be adjusted in the y-axis translation, the z-axis translation, and the x-axis rotation. The seat height, the seat depth, and the pedal length can also be adjusted. Thus, the experimental wheelchair is fully adaptable for different sitting postures and can accommodate people of different body sizes. The back support and cushion of experimental wheelchair was equipped with soft rubbers, which provided participants with smooth surfaces, thereby reducing the potential discomfort that results from concentrated stress at the contact area.

### Experimental Protocol

When being seated in the experimental wheelchair, the arms of each participant were relaxed at the side of their body. The thighs were roughly parallel to the floor while the feet were rested firmly on a pedal with a 65-degree hanger angle and positioned the shoulder width apart.

**Figure 2:**
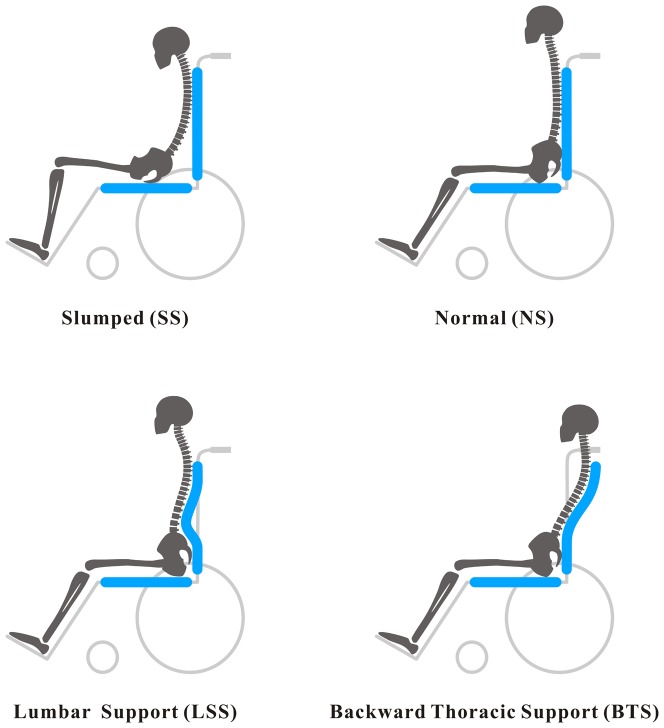
Four sitting postures. Including the slumped (SS), normal (NS), lumbar support (LSS), and backward adjustable thoracic support (BTS) sitting postures.

As shown in Figure 2, the SS, NS, LSS, and BTS were achieved respectively by using different configurations on the experimental wheelchair and the participants' body movements. Relating to wheelchair configurations, for the SS and NS postures, the back support remained flat. In the LSS posture, the lumbar support was adjusted to the prominent 4 cm width at the L3 of the lumbar vertebra of each participant [20], [21]. For BTS posture, the chair was configured so that the thoracic support was adjusted backward for 8 cm and located at T7–T12 of the thoracic vertebra. About the participants' body movements, under the SS posture condition, the participants were instructed to position the pelvis in the middle of the seat, allowing it to significantly tilt posteriorly, with the trunk maintaining a C-shaped posture against the backrest [3]. While the NS, LSS and BTS postures, the participants were instructed to rest their buttocks as far back in the seat as possible, and to have the back flat against the backrest [21].

All the participants were asked to practice the postures until they could easily perform all of them. The posture test sequence was randomized, and each sitting posture was maintained for 5 seconds.

### Spinal Curvature Measurement

**Figure 3:**
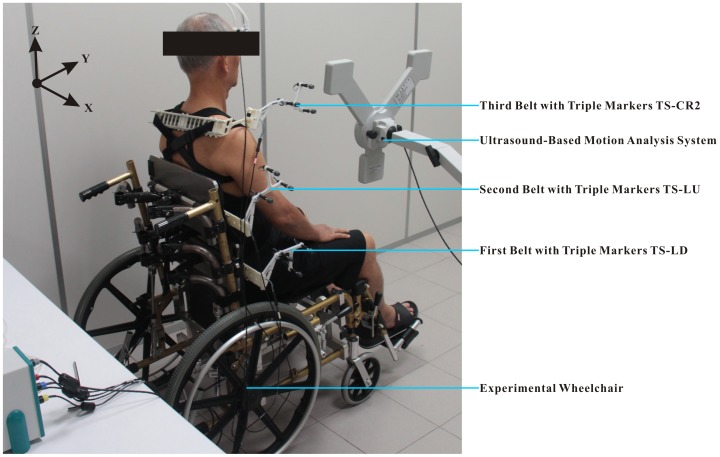
Experimental setup. The picture shows the experimental setup with experimental wheelchair, ultrasound-based motion analysis system, and miniature ultrasonic modules. The first belt with triple markers TS-LD was situated around the level of the posterior superior iliac spines and the anterior superior iliac spines. The second belt with triple markers TS-LU was placed firmly around the T12 level. The third belt with triple markers TS-CR2 was placed firmly around the T1 level.

**Figure 4:**
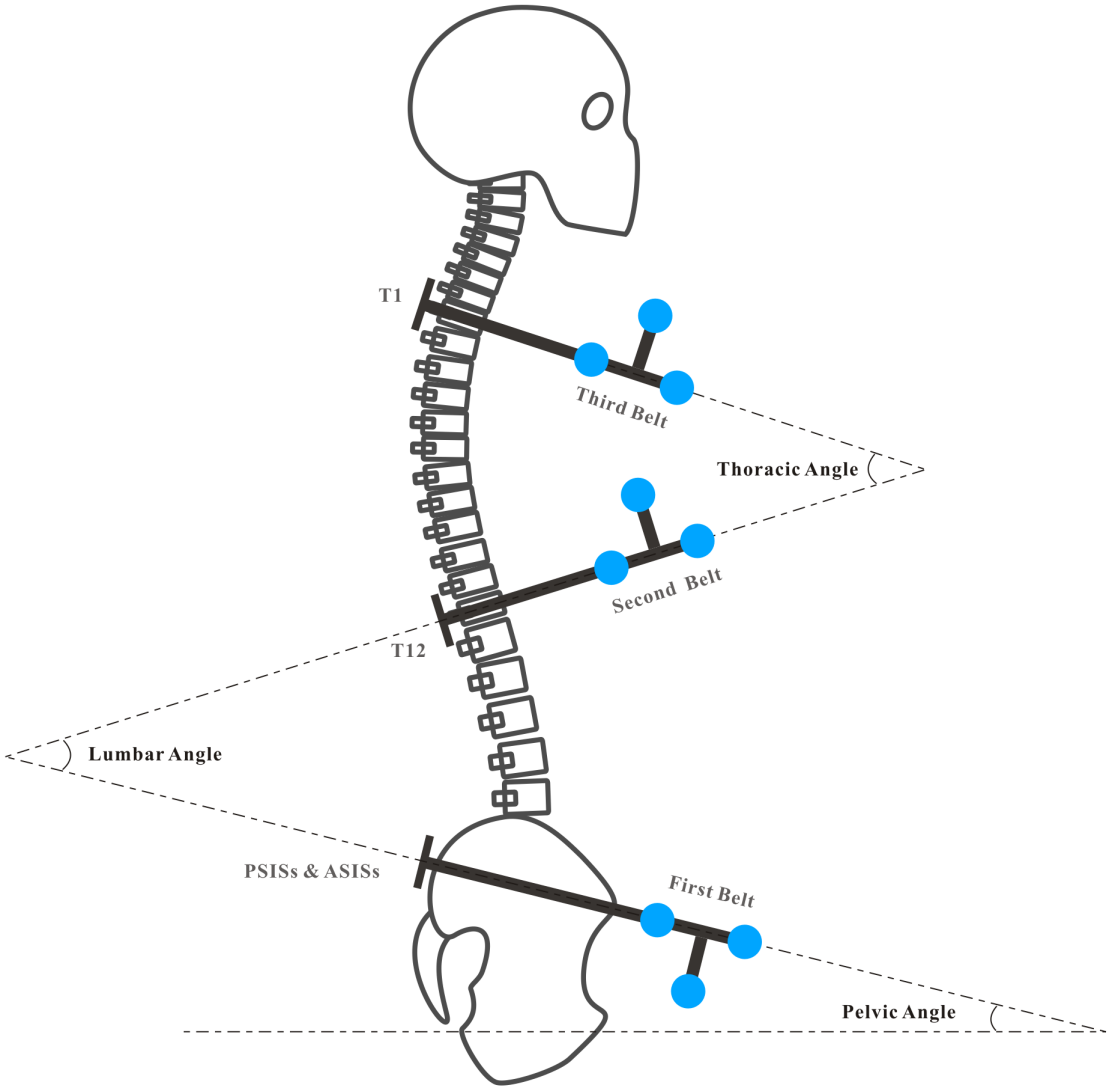
The back attachment placement and angle definition. The angular values of spinal regions were then defined for the following: pelvic (the first belt (PSISs & ASISs) relative to the horizontal line), lumbar (the second belt (T12) relative to the first belt (PSISs & ASISs)), and thoracic (the third belt (T1) relative to the second belt (T12)).

Spinal curvature was recorded by using an ultrasound-based motion analysis system (CMS20S Measuring System; zebris Medical GmbH, Isny im Allgäu, Germany). It has shown high levels of reliability [22], [23]. Data were sampled at the frequency of 25 Hz. The pelvic, lumbar, and thoracic angles were measured by using back attachment accessories (Attachment set with triple markers TS-LD, TS-LU, and TS-CR2; zebris Medical GmbH, Isny im Allgäu, Germany) [24]. The accessories consist of distinctively designed belts, each of which is attached to a series of 3 miniature ultrasound transmitters that are fixed to a participant's trunk (Figure 3). Before conducting the spinal curvature measurement, all of the back attachment accessories were neatly arranged and fixed on a vertical frame, and then calibrated to zero by CMS20S Measuring System. The first belt with triple markers TS-LD was situated around the level of the posterior superior iliac spines (PSISs) and the anterior superior iliac spines (ASISs). The second belt with triple markers TS-LU was placed firmly around the T12 level. The third belt with triple markers TS-CR2 was placed firmly around the T1 level. The angular values of spinal regions were then defined for the following: pelvic (the first belt relative to the horizontal line), lumbar (the second belt relative to the first belt), and thoracic (the third belt relative to the second belt), as illustrated in Figure 4. The positive value (+) represents the posterior tilt or kyphosis while the negative value (−) represents the anterior tilt or lordosis. Spinal curvatures were calculated by using the WinData software (WinData, version 2.22.25; zebris Medical GmbH, Isny im Allgäu, Germany).

### Back Muscle Activation Measurement

Back muscle activation was recorded at a sampling rate of 1000 Hz by using an 8-channel surface electromyography (EMG) system (The zebris EMG Bluetooth Measuring System; zebris Medical GmbH, Isny im Allgäu, Germany), with a built-in preamplifier (gain  = 1000). The EMG system bandwidth was 7 bits 500 Hz, and the common mode rejection ratio was 110 dB.

Before the EMG measurement, in order to reduce skin impedance, the skin of electrode site was cleaned with alcohol, shaved with a razor, and lightly abraded with a fine sandpaper. Pairs of self-adhesive Ag-AgCl surface electrodes (Medi-trace 100 series; Covidien, Massachusetts, United States) with an inter-electrode distance of 2.5 cm were placed in parallel to the test muscles on both sides. The electrodes were taped on superficial lumbar multifidus (LM: L5 level, parallel to a line connecting the PSIS and L1–L2 interspinous space) [25], lumbar erector spinae (LES: 3 cm lateral to the L3 spinous process) [26], iliocostalis lumborum pars thoracis (ICLT: level of the L1 spinous process, midway between the midline and lateral aspect of the participant's body) [27], and thoracic erector spinae at the T9 level (TES: 5 cm lateral to the T9 spinous process) [26] on the left (-L) and right (-R) side. A reference electrode was placed on the right iliac crest.

For the normalization of EMG data, the aforementioned muscles were normalized to maximal voluntary isometric contraction (MVIC). The full details of these normalization procedures have been outlined in highly reliable studies [27]–[29].

The raw EMG data were processed by using the customized software program MATLAB (MATLAB, version7.7; The MathWorks, Massachusetts, United States). The raw data were first checked and removed for the heartbeat and other artifacts by using a customized program in MATLAB. Then, the raw data were demeaned, full wave rectified, filtered by using a fourth-order Butterworth filter with a cut-off frequency of 4 to 400 Hz, and a 25 ms moving window was applied to the linear envelope [28].

### Statistical Analysis

The Statistical Package for the Social Sciences (SPSS, version 21; IBM North America, New York, United States) was used for conducting all statistical analyses. All parameters, including spinal curvatures (pelvic, lumbar, and thoracic angles) and back muscle activations (MVIC of the LM-L, LM-R, LES-L, LES-R, ICLT-L, ICLT-R, TES-L, and TES-R) were compared among the four different sitting postures by using a one-way analysis of variance with repeated measures. The Tukey's post hoc test (if Levene's test was not significant) or Games-Howell post hoc test (if Levene's test was significant) was used for detecting statistically significant differences in the dependent variables across the tests. The statistical significance was set at *P*<0.05.

## Results

All the participants completed the spinal curvature and back muscle activation measurements using the experimental wheelchair in the SS, NS, LSS, and BTS postures. None reported adverse reactions to the experimental protocol.

### Spinal Curvature

**Figure 5:**
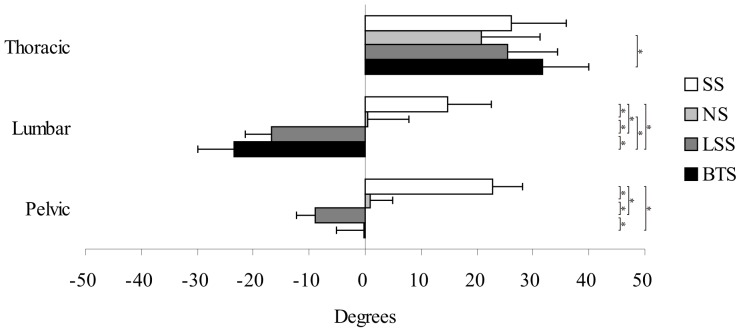
The results of spinal curvature measurement. Comparison of mean pelvic, lumbar, and thoracic angles across 4 sitting postures, which include the slumped (SS), normal (NS), lumbar support (LSS), and backward adjustable thoracic support (BTS) sitting postures. The positive value (+) represents the posterior tilt or kyphosis while the negative value (-) represents the anterior tilt or lordosis. Error bars indicate SD and * indicates p<0.05.

The results of the spinal curvature measurements are shown in Figure 5. In contrast with the SS posture, the NS, LSS and BTS postures appeared to yield significantly lower pelvic and lumbar angle values (*P*<0.001). When compared with the NS posture, the LSS posture appeared to yield significantly lower pelvic and lumbar angle values (*P*<0.001). In contrast with the NS posture, the BTS posture appeared to yield significantly lower lumbar angle values (*P*<0.001), but no significant differences in pelvic angle were observed. The BTS posture appeared to yield significantly higher values for pelvic angle (*P*<0.001) and lower values for lumbar angle (*P* = 0.014) when compared with the LSS posture. No significant differences in thoracic angle were observed, except for the NS versus the BTS, The latter posture appeared to yield significantly higher values (*P* = 0.003).

### Back Muscle Activation

**Figure 6:**
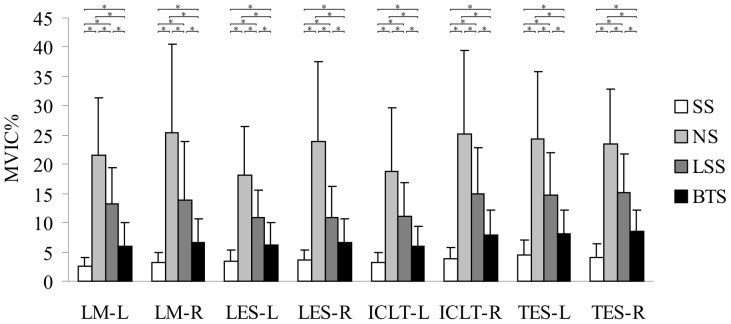
The results of back muscle activation measurement. Comparison of mean maximal voluntary isometric contraction (MVIC) of the lumbar multifidus (LM), lumbar erector spinae (LES), iliocostalis lumborum pars thoracis (ICLT), and thoracic erector spinae at T9 (TES) across 4 sitting postures, which include the slumped (SS), normal (NS), lumbar support (LSS), and backward adjustable thoracic support (BTS) sitting postures. Left side indicate -L, Right side -R, Error bars indicate SD, and * indicates p<0.05.

The results of the back muscle activation measurements are shown in Figure 6. In contrast with the SS posture, the NS, LSS and BTS postures appeared to produce significantly more muscle activity in all the tested muscles (*P*<0.05). Compared with the NS posture, the LSS and BTS postures appeared to produce significantly less muscle activity in all the tested muscles (*P*<0.05). Compared with the LSS, BTS posture appeared to produce significantly less muscle activity in all the tested muscles (*P*<0.05).

## Discussion

Choosing a suitable wheelchair seating system is essential for patients with lower limb disorders for improving postural support and decreasing the risk of complications during the rehabilitation phase or daily life. This study suggested a new sitting posture concept, which is the BTS posture. We also quantitatively investigated the biomechanical effects induced by the BTS posture and compared it with 3 common wheelchair sitting postures. The BTS posture was defined by a rather neutral pelvic tilt, high lumbar lordosis and low back muscle activation. Consequently, the results confirmed our hypothesis.

Pelvic tilt may influence hamstring tightness [11], [12]. In this study, the SS posture presented the greatest posterior pelvic tilt among the 4 sitting postures, decreasing hamstring tightness. However, the posterior pelvic tilt increased the shear stress between the sacral spine and the seat, thereby increasing the risk of sacral pressure ulcers [30]–[32]. The LSS posture exhibited the greatest anterior pelvic tilt, increasing hamstring tightness. In addition, the muscles were tensed because of stretching, which increased the passive tension load of peripheral viscoelastic tissues and the mechanical microvascular resistance and reduced the capillary diameter, the blood flow, and the intracapillary red blood cell velocity [9], [33], [34]. These changes may influence the nutrition metabolism of surrounding tissues. A compromising and balanced method through the NS and the BTS postures could enable us to have a neutral pelvic tilt.

Lumbar curvature could influence the stress on the intervertebral discs [9]. We found that the SS posture exhibited the greatest level of lumbar kyphosis among the 4 sitting postures. It could increase stress and metabolite accumulations on the intervertebral discs, causing disc degeneration and herniation [9], [35]. The NS posture appeared to generated a relatively flat lumbar curvature. Insufficient lordosis cannot effectively reduce the hydrostatic pressure in the nucleus pulposus of the intervertebral disc [9]. In addition, patients with flat lumbar may experience problems of nonfunctioning absorption mechanism [36]. Both the LSS and the BTS generated lumbar lordosis, which can transfer the force exerted on the lumbar to the inferior margins of articular surfaces of zygapophysial joints. As a result, the stress-shielding effect will reduce intervertebral disc stress [9]. The difference between the LSS and the BTS is that latter can generate greater lumbar lordosis. In addition, the lumbar lordosis resulting from the LSS posture is likely to be compensated by an anterior pelvic tilt while the BTS posture provides a compensation for thoracic kyphosis.

Regarding thoracic angle, the results showed that the NS and the BTS posture exhibited significant differences. The latter exhibited the most kyphotic among the 4 sitting procedures. However, the measured values were within the normal kyphosis range found in the literature (20°–50°) [37]. The fact that the BTS posture may influence pulmonic functions requires further investigation.

Several studies have suggested that increasing the back muscle activity may generate negative effects [9], [38], [39]. In this study, the results showed that the SS posture involved the least muscle activity among the 4 sitting postures. During the experiment, most participants perceived that the SS posture was the most relaxing and comfortable. Previous studies have indicated that the muscle strength and mass decreases with age [40], [41], which influences posture maintenance. Muscle weakness could explain why the wheelchair-bound elderly adults often adopt the SS posture. The SS posture generates a flexion-relaxation phenomenon characterized by trunk stabilizers that change from active (the muscles and tendons) to passive (the intervertebral discs, ligaments, fascia, and vertebral bones) spinal structures, thereby increasing the risk of back pain [42], [43]. Of the 4 sitting postures, the NS posture involved the most muscle activity. Previous research indicated that a prolonged and continuous muscle contraction that exceeds approximately 20% of MVIC will cause a lack of oxygen, the accumulation of sour metabolites, an intracellular shortage of potassium, the production of pain, and generate muscle spasms [38]. Six back muscles for which the MVIC were more than 20% in the NS exist, which are the LM-L, LM-R, LES-R, ICLT-R, TES-L, and TES-R. When compared with the NS and the LSS posture, the BTS generated lower muscle activity. A possible cause of this result is that the parts of back muscle load for maintaining the body posture and weight was transferred to the BTS back support. The actual load transference requires further confirmation. In addition, the muscle activity observed in this study tended to be high. A possible cause could be that the degenerating back muscles of the elderly adults influenced the MVIC ratio.

In study limitations, the participants in this study were the healthy elderly adults, rather than wheelchair patients with lower limb disorders. Future studies including actual wheelchair-bound patients are suggested. In addition, this study only focused the short-term evaluations. Long-term effects must be further verified.

Age-related muscle strength degeneration influenced the maintenance of postures. The results of this study showed that the BTS posture could maintain a neutral pelvic tilt and lumbar lordosis through a lower back muscle load, thereby indirectly reducing the hamstring tightness and stress on the intervertebral disc. A further examination on the back pain relief through the BTS sitting posture to needed.

## Conclusions

Considering the continuously degenerative physiological structure of the wheelchair-bound elderly people, the BTS concept was suggested because it generates a neutral pelvic tilt, high lumbar lordosis, and lower back muscle activation. The results of this study contribute to the decision-making processes of wheelchair seating systems for consumers, clinicians, and manufacturers. Further research is necessary to discover the optimal backward degree for BTS and the optimal period for regulating the backward and forward alternation of the BTS during prolonged sitting.
